# Endocrine-Disrupting Chemicals and Their Adverse Effects on the Endoplasmic Reticulum

**DOI:** 10.3390/ijms23031581

**Published:** 2022-01-29

**Authors:** Kangmin Kim, Jin-Sook Kwon, Changhwan Ahn, Eui-Bae Jeung

**Affiliations:** 1 Laboratory of Veterinary Biochemistry and Molecular Biology, College of Veterinary Medicine, Chungbuk National University, Cheongju 28644, Korea; jfun4my@gmail.com (K.K.); gonkjs@gmail.com (J.-S.K.); 2 Laboratory of Veterinary Physiology, College of Veterinary Medicine, Jeju National University, Jeju-si 63243, Korea; cahn@jejunu.ac.kr

**Keywords:** endocrine system, endocrine-disrupting chemical, endoplasmic reticulum, unfolded protein response

## Abstract

There is growing concern regarding the health and safety issues of endocrine-disrupting chemicals (EDCs). Long-term exposure to EDCs has serious adverse health effects through both hormone-direct and hormone-indirect ways. Accordingly, some EDCs can be a pathogen and an inducer to the susceptibility of disease, even if they have a very low affinity on the estrogen receptor, or no estrogenic effect. Endoplasmic reticulum (ER) stress recently attracted attention in this research area. Because ER and ER stress could be key regulators of the EDC’s adverse effects, such as the malfunction of the organ, as well as the death, apoptosis, and proliferation of a cell. In this review, we focused on finding evidence which shows that EDCs could be a trigger for ER stress and provide specific examples of EDCs, which are known to cause ER stress currently.

## 1. Introduction

### 1.1. Brief Status of EDC

Since 1990, Colborn et al. introduced the term ‘endocrine disruptor’ and issued a consensus statement regarding the adverse effects of EDCs on humans and environmental health [1]. There are many EDC definitions. Among them, the World Health Organization (WHO) and the United States Environmental Protection Agency (EPA) definitions are the most comprehensive and official. There is some commonality between the definitions of the EDCs of the WHO and the United States EPA. The definition by the WHO in 2002 states that “an EDC is an exogenous compound or mixture that alters the function of the endocrine system and, consequently, causes adverse health effects”. The definition by the EPA is “an EDC is an exogenous compound that may interfere with the synthesis, secretion, transport, metabolism, receptor binding, or elimination of endogenous hormones, altering the endocrine and homeostasis system.” Both dictate the heterogeneity of EDCs and the disruption of the normal endocrine system. On the other hand, the understanding of the mechanisms through which EDCs exert their adverse effect has broadened. Most EDCs are artificially synthesized substances that interfere with the hormone-binding receptors, such as nuclear receptors, non-nuclear hormone receptors, and numerous other hormonal receptors that affect the endocrine and reproductive systems [2,3]. Thus, EDCs disrupts the hormonal and homeostasis system of an organism.

### 1.2. EDCs and Related Disease

Humans are exposed to EDCs through various routes: inhalation, skin contact, food uptake, and collateral uptake of animals contaminated by EDCs [4]. The collateral uptake indicates the long half-life of EDCs, which explains the frequent co-contamination by EDCs [5]. Early-in-life exposure results in later-in-life diseases, such as reproductive/endocrine diseases [6,7,8], immune diseases [9], cardiopulmonary diseases [10], the brain/nervous system disease [11], and cancer [12,13].

In particular, in animal models [14] and in human epidemiological studies [3], it was found that exposure to EDCs at an early stage of development often leads to fatal diseases, such as endocrine-related cancer or neurodevelopmental diseases, such as ADHD and autism. There are some excellent examples of the adverse effects of EDCs in the authors’ laboratory. Bonn Lee and colleagues reported the synergistic effects of the developmental neurotoxicity of the combined exposure to the EDCs nicotine, diazinon, and organophosphate [15]. Tran Nam et al. reported that maternal exposure of octamethylcyclotetrasiloxane caused abnormal neurodevelopment and behaviors [16].

Higher urinary BPA concentrations were associated with cardiovascular diagnoses in age-, sex-, and fully-adjusted models (OR per 1-SD increase in BPA concentration, 1.39; 95% confidence interval (CI), 1.18–1.63; *p* = 0.001 with full adjustment). Higher BPA concentrations were also associated with diabetes [17].

The purpose of this review is to provide pathway to clarify the link between basal molecular mechanisms, in particular, ER stress and EDC exposure-induced pathophysiology.

## 2. ER Stress and EDCs

### 2.1. The Link between EDCs and ER Stress

There is no clear mechanism of how EDCs cause endoplasmic reticulum (ER) stress. On the other hand, ER stress markers are a representative signal for the adverse effects of EDCs. ER stress activates a signaling network called the unfolded protein response (UPR) to restore ER homeostasis through discarding the stress cause, such as a misfolding protein. However, under prolonged and severe ER stress, the UPR can become cytotoxic (including apoptosis) rather than cytoprotective [18]. There are several UPR activating processes that retain the homeostasis of protein folding. The accumulation of reactive oxygen species (ROS) or the perturbation of cellular compositions, such as lipid and cholesterol, activate the UPR [19,20,21]. Interestingly, almost kinds of EDCs showed an elevation of ROS. Both oxidative stress and EDC exposures have been associated with metabolic syndromes, insulin resistance, diabetes, obesity, and cardiovascular complications [17,22].

### 2.2. ER Stress

The vast majority of studies used ER transmembrane genes, such as Grp78, also called heavy chain binding immunoglobulin proteins (BiP), PKR, such as endoplasmic reticulum kinase (PERK), inositol-requiring 1 protein α (Ire1α), activating transcription factor 6 (ATF6), and their down-regulated genes, as typical markers because they react sensitively upon the accumulation of the UPR, the mechanism of which will be discussed in the next section.

**Figure 1:**
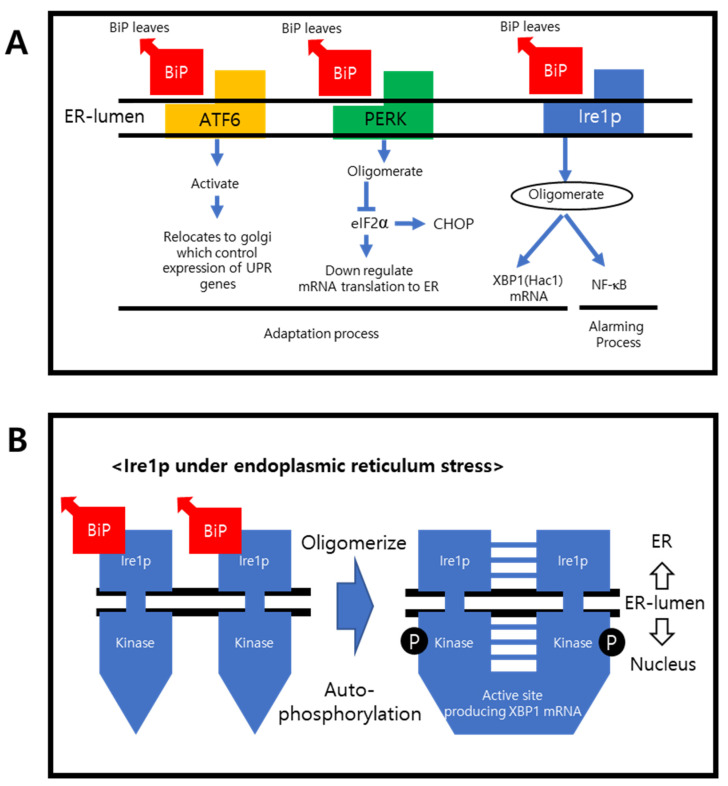
ER stress regulating genes. (**A**) Schematic flow of the transmembrane UPR alarming genes and the process of how they react upon UPR is shown. Upon sensing UPR, BiP leaves, and transmembrane protein phosphorylates and oligomerizes. Then they go through the adaptation process. (**B**) After the BiP leaves the transmembrane proteins, such as Ire1α, it then oligomerizes on the nucleus side of carboxy terminus, and XBP1 mRNA is activated.

Figure 1A shows the transmembrane proteins and their downregulating pathways. In their normal states, Grp78/BiP are attached to the transmembrane proteins, preventing them from oligomerizing. During the adoptive and alarming sequences under ER stress, BiP detaches primarily from the transmembrane proteins and binds to the unfolded proteins. The transmembrane proteins are activated by oligomerization and regulate the targeted ER stress compensating genes.

PERK is also activated after BiP detachment. PERK inhibits eukaryotic translation initiator factor 2α (elF2α) within the nucleus, down-regulating mRNA translation to the ER [23]. Upon activation, ATF6 is relocated to the Golgi and sends a signal to regulate the expression of the UPR genes. The endonuclease-containing carboxy end is then activated and upregulates the endonuclease chaperones, foldase, and the clearance rate. The misfolded or incorrectly assembled proteins will be eliminated by ERAD [24]. If this adoptive process fails, the cell undergoes apoptosis, which is regulated by the CHOP. Oligomerized Ire1α can activate the UPR alarming gene, nuclear factor-kB (NF-kB), to produce XBP1 mRNA, which upregulates specific ER-resident chaperones [25,26]. The X-Box binding protein 1 (XBP1) has two isoforms, where XBP1 is spliced and XBP1 is unspliced. Spliced XBP1 is a transcriptional factor that regulates the transcription of the UPR gene, whereas the unspliced XBP is an inactivated form [27,28]. Hac1 is the yeast name of XBP1.

## 3. Specific Examples of EDCs Affecting the ER

This chapter encompasses the ER that affects EDCs and their pathological roles. Table 1 provides examples of some EDCs which give rise to ER stress. These chemicals are commonly encountered. Most of them are endocrine disruptors. The ER stress was measured mainly by Western blotting, PCR, and mass spectrometry [29]. The detected proteins were GRP78/BiP, ATF6, PERK, and Ire1α. These proteins increased compared to the control group.

The compounds were grouped by four categories: the name of the chemicals, their uses, classifications, and if they are an upregulating ER stress regulator. Normally, the EDCs are used as additives for certain products such as flame retardants, herbicides, and plastics. Their classification, in this regard, states the disease which could be caused by the chemical. The ER stress regulator presents the markers the chemicals upregulate, which are supported by multiple citations. Regarding the classifications, different types of EDCs have adverse effects on different parts of the organ and system. For example, bisphenol A has adverse effects on spermatogenesis, whereas atrazine is potentially toxic to the immune system. These aspects are included in the Table 1 classification; however, we were not able to pin down one organ or system, for example, as stated in Part 3.9, where glyphosate which exerts multiple toxic effects on multiple organs and systems was classified as a reproducible toxicant since the main literature stated glyphosate as being a reproducible toxicant.

### 3.1. Atrazine

Atrazine is used as an herbicide and is a well-known biohazard molecule. Atrazine can be introduced to the human body through various pathways, such as inhalation, and its dermal absorption during application. There are many reports on atrazine as an EDC [76,77] and its immunotoxic effects [9]. Lee et al. reported that the oral administration of atrazine reduced the T-lymphocytes in mice and induced programmed cell death. Subsequently, the ER stress markers GRP78, eIF2α, p-EIF2α, and CHOP were detected [32]. It is reported that atrazine exerts a hepatotoxicity effect on the detoxification enzymes and increases a relative liver weight (liver weight(g)/body weight(g)) at certain atrazine concentrations, which is closely related to hepatocyte lipid accumulation. Subsequent ER stress marker ATF6 and GRP78 were detected in the quail liver [30]. Furthermore, Tchounwou et al. reported that atrazine alone did not affect human liver carcinoma cell HepG_2_ significantly, so they worked on the combined effect of atrazine and arsenic trioxide. The results showed that the combined chemical exerts significant ER stress on the HepG2 cell [33]. Atrazine also upregulated ATF6 and PERK in murine splenocyte models [31].

### 3.2. Bisphenol A and Their Metabolites

BPA is a phenolic-type compound that is used widely in industry of plastics. BPA is a well-known EDC with versatile modes of action [78], especially its adverse effect on the male reproductive system [34]. Upon exposure to BPA, the ATF6 pathway is upregulated in the murine endothelium [36], the murine neuroblastoma cell line [37], mouse spermatocytes [38], the livers of ovariectomized mice [35], and the human breast epithelial cell line, MCF-10F [42]. PERK and downstream pathways were up-regulated for sheep gestational adipose tissue [41], the murine endothelium [36], neuro-2a murine neuroblastoma cells [37], mouse spermatocytes [38], the BPA-treated paternal mouse offspring’s myeline sheath [44], the human breast carcinoma cell VM7Luc4EZ [42], the adult mouse hypothalamic cell line [40], and mouse nonparenchymal hepatocytes [43]. Ire1α pathway is upregulated in sheep gestational adipose tissue [41].

Among them, a few noteworthy examples will be discussed. BPA is known as an obesogen. Obesogens are a classification of EDC which interferes with lipid metabolism, metabolic sensors, and energy balance [79]. Figueiredo et al. explored the BPA’s effect of lipid accumulation on the livers of ovariectomized mice, which showed the lipid metabolic dysfunction effect [35]. Pu et al. investigated the relationship of ER stress in modulating the adipogenic potential of mice gestationally exposed to BPA and its analogue, bisphenol S. Since ER stress can modulate adipogenesis, the relative mRNA expression for the ER stress markers Ire1α and PERK were detected [41]. Toledano et al. tried to evaluate the mechanisms that are involved in BPA-induced cell aging and the related ER stress on murine aortic endothelial cells. By Western blot methods, they confirmed that the proteins concerning the cell aging was up regulated. Furthermore, it was experimentally proved that upon exposure to BPA, a subsequent superoxide was produced, which generates the oxidative stress and, thus, activates the UPR. Conclusively, cell aging is accelerated [36]. Junpei et al. studied BPA-induced liver injury without the involvement of estrogen receptors [43]. BPA is a known liver toxicant [17], and it is assumed that BPA would bind to the estrogen receptor and affect the target cells. However, the mechanism was unclear. On the other hand, in 2003, Kabuto et al. reported that BPA could disturb the cell resistance against reactive oxygen species (ROS) [80]. Therefore, the BPA-associated ROS could be the key initiator for hepatotoxicity. Junpei et al. used NCTC clone 1469 mice’s non-parenchymal hepatocytes. The results showed no mitochondrial damage or depletion, but ER stress was observed [43]. Several markers for ER stress were measured, and the morphological alteration of the ER was also observed. The cells were co-treated with estrogen receptor inhibitors and BPA to determine if the estrogen receptors were involved in BPA-induced liver cell damage. Interestingly, the inhibitors could not stop the apoptosis of the liver cells, because the decrease in hepatocyte viability could be alleviated using a ROS scavenger. Hence, the source of ER stress and cell death can be attributed to ROS. The metabolite of BPA could damage the cell [81]. 4-Methyl-2,4-bis(4-hydroxyphenyl)pent-1-ene (MBP) was discovered to be a metabolite of BPA [81]. Several studies have shown that BPA is harmful to lung functions [82,83], and the collateral damage induced by the metabolites of BPA was studied. MBP was reported to have a higher affinity to the estrogen receptors α and β than BPA [84]. The ER is a particularly important organelle in type 2 alveolar cells in the lung because it synthesizes the surfactant protein [85]. Hence, ER stress has a direct relationship with type 2 alveolar function and might cause lung dysfunction [81]. Paternal exposure to BPA affects the next-generation prefrontal cortex. Accordingly, an increase in abnormal behavior was measured [44]. It has a straightforward relationship because the influence from the father is only from the sperm donation. The study showed that the offspring of the paternal mouse exposed to BPA for 10 weeks showed ER stress in the prefrontal cortex. Because the prefrontal cortex governs anxiety and sociability, a behavior test was also carried out to support the result. The behavior test result was concordant with the former proteomic research result.

### 3.3. Butylparaben

Butylparaben (BP) is used as preservative of cosmetics, foods, and chemical. It is a known EDC and exerts an estrogenic effect [86] and is a reproductive toxicant [6,7]. Research by Yang et al. explored the adverse effect of BP on human trophoblast cells. The results showed increase in GRP78, p-eIF2α, ATF6, and Ire1α [47].

### 3.4. Chlorpyrifos

Chlorpyrifos is a widely used organophosphorus pesticide and exposure to chlorpyrifos could induce Alzheimer’s disease and Parkinson’s disease [48]. Reyna et al. observed oxidative stress in JEG-3 cells upon chlorpyrifos exposure. In their study, chlorpyrifos was experimentally proven to induce ER stress, which was elucidated by increased p-eIF2α [49]. Furthermore, Reyna demonstrated that chlorpyrifos destabilized p53 tumor suppressors in JEG-3 cells. This work was performed to investigate the major mediator of these pathways. Since p53, the tumor suppressor protein, plays a major role in the response to cellular stress, p53 was affected by chlorpyrifos exposure [49]. Moreover, Anderson et al., in their study, tried to evaluate the cellular mechanisms of chlorpyrifos toxicity in neurons, since there are few reports on how organophosphates are directly related to neurodegenerative diseases [48,87,88,89]. In the paper, Anderson et al. tried to characterize the mechanism of chlorpyrifos neurotoxicity, and the results showed that ER stress was induced by chlorpyrifos, as well as the suppression of Bcl2 binding component 3, which helps cells to survive [48].

### 3.5. Dibutyl Phthalate

Dibutyl phtalate (DBP) is an environmental EDC which is widely used in the polyvinyl chloride (PVC) industry and as a personal care product. Prolonged exposure to DBP during the developmental stage can lead to obesity in adulthood [50,51,90]. Li et al. examined the effect of DBP exposure during the fetal stage on obesity. They focused on the intrauterine exposure of DBP. They did not report the exact mechanism of DBP exposure and ER stress, but the relative RNA levels of BiP and CHOP increased significantly. In addition, the bodyweights of 15–21-week-old postnatal mice were significantly greater. This result could be explained by its interference with the obesity indicators, such as leptin, cholesterol, and triglyceride [55]. DBP also induce oxidative stress in the germ cell, hence causing adverse effects on the reproductive system, as well as up-regulating ATF6, the PERK downstream pathways, and GRP78 [52]. Another experiment by Zhang et al. used mouse spermatocyte derived GC-2spd cells and the results showed an increase in p-PERK [54]. The effect of in vivo DPB exposure, during fetal development, on obesity was studied, and GRP78 and CHOP were upregulated [55].

### 3.6. 2,4-Dichlorophenol

2,4-Dichlorophenol (2,4-DCP) is a widely used herbicide and it is a known EDC [8]. Zhang et al. used mEF cell lines to explore the potential mechanisms of the ER stress pathway under the influence of 2,4-DCP. The result used Ire1α, ATF6, GRP78, and CHOP as markers for ER stress [56]. Furthermore, the chemical was used to evaluate hepatotoxicity on the human liver cell, HL7702. Upregulated GRP78, CHOP, and p-eIF2α were detected [57].

### 3.7. Dichloro Diphenyl Trichloro Ethane

Dichloro diphenyl trichloro ethane (DDT) is a banned pesticide; however, DDT and its metabolites still remain in the environment [91] and are a threat to living organisms [92]. Pavlikova et al. explored the acute toxicity of DDT exposure in pancreatic beta cells and showed an up-regulation of CHOP and GRP78 upon prolonged exposure [58].

### 3.8. Di (2-Ethylhexyl) Phthalate

Among the various types of phthalate compounds, di(2-ethylhexyl)phthalate (DEHP) is the most commonly used. It is used as a plasticizer and is commonly found in the environment, such as in various medical devices, food containers, and children’s toys [93]. On the other hand, DEHP does not bind tightly to plastics, so it can easily migrate into the surroundings [94]. DEHP has been reported to potentially cause type II diabetes [59]. Upon exposure to DEHP, ATF6 and Ire1α are up-regulated in the female quail kidney [60] and the testes of offspring from DEHP-exposed mice [61]. The PERK downstream signalling upregulation was observed for quail kidneys [60], the testes of mouse offspring [61], the rat insulinoma INS-1 cell [62], the human neuroblastoma SH-5Y cell [63], monkey kidney vero cells, and human keratinocytes [64]. GRP78 was also up-regulated for all above stated references.

It has been pathologically reported that DEHP caused severe reproductive toxicity in the human body [95,96]. In addition, DEHP can cross the placental barrier easily [97] and pass into maternal milk [98], causing fatal damage to the offspring [99]. In male rodents, male reproductive injuries caused a reduction in testicular weight, spermatogenesis, and could even cause infertility [100,101,102]. Electron microscopy revealed the relationship between the ER lumen and DEHP. DEHP swells the ER lumen, which disables the biosynthesis folding assembling secreted protein [61]. DEHP has a close correlation with type 2 diabetes [103,104]. An animal study also reported a similar result, where a certain dose of DEHP disrupted the insulin signal [105,106]. Similar to BPA, DEHP may induce oxidative stress and generate ROS [107], which modulates the ER stress signaling pathway.

A dysfunction of ER stress-mediated pancreatic Bcells play a key role in type 2 diabetes, because the ER is an essential organelle for insulin biosynthesis. Therefore, it is important to look into the ER stress markers of pancreatic B-cells. Xia sun et al. provided close insight regarding this matter [62]. They treated rat insulinoma INS-1 cells with DEHP. The INS-1 cells were used because they are susceptible to oxidative stress due to their low oxygen radical scavenger content [108]. The cellular levels of insulin, INS-1, ROS level, and ER stress markers were measured. At a certain DEHP concentration, insulin secretion from INS-1 cells was reduced significantly, and DEHP-stimulated ROS production was also confirmed by a fluorescence assay, confirming ER stress. The metabolites of DEHP were reported to be mono(2-ethylhexyl)phthalate (MEHP); however, MEHP does not penetrate the plasma membrane as severely as DEHP [109].

### 3.9. Glyphosate

The most widely used herbicide, glyphosate, is a reproductive toxicant [65] and exerts adverse effects on a variety of organs and system [110,111]. It is also a suspected reproductive toxicant, and to prove its toxicity, Xia et al. confirmed its effect and mechanism on testosterone synthesis and secretion in mouse TM3 cells. Glyphosate inhibits the expression of StAR and CYP17A1, the testosterone synthase, therefore reducing the testosterone synthesis. The testosterone synthase decrement was dependent on the PERK/eIF2α mediating pathway, which was closely interrelated to the decrement. The relationship was checked using the PERK inhibitor GSK2606414 [66].

### 3.10. Lead

Lead is a known toxicant and causes unhealthy effects in living organisms [112]. Chronic exposure to lead could cause adverse effects on every aspect, such as cancer [13] and Parkinson’s disease [67]. The study by Gao K et al. explored the cytotoxicity of lead and its mitophagy [68]. The experiment used HEK 293 human embryonic kidney cells and induced mitophagy by lead. The results showed that lead caused mitophagy, as well as ER stress, by up-regulating ATF6, Ire1α, CHOP, and GRP78 in HEK293 cells. Furthermore, Gao et al. attempted to work out the relationship between the mitophagy and ER stress by using 4-PBA, the ER stress inhibitor. The result showed a significant decrease in the degeneration of the mitochondria marker protein, which suggests that ER stress and mitophagy have close relationships with each other [68,113]. Qian et al., in their study, used rat astroglia both in vivo and in culture and experimentally detected an upregulation of GRP78 [69].

### 3.11. 4-Nonyl Phenol

4-Nonyl phenol(4NP) is an environmental pollutant from industrial surfactants. 4NP was reported to be an EDC because it can bind to estrogen receptors and interfere with the human metabolism [114].

Regarding the influence of 4NP, studies detected CHOP upregulation in rat primary cortical neurons [71], human liver HepG2 cells [72], and PC12 cells [73]. Moreover, GRP78 is upregulated in every reference [70,71,72,73,74,75]. Several disease models have been reported. 4NP exerts its effect on human fertility [115], oxidation stress, and mitochondria dysfunction in the pancreas [116]. On the other hand, mainly gastrointestinal diseases have been reported, because 4NP is ingested mainly from contaminated food and water. The intestine is the first organ to be damaged by the EDC because the gastrointestinal route is the main 4NP uptake route. Lepretti et al. examined intestinal cell cytotoxicity and ER-related apoptosis [70]. The adverse effects were categorized first. The epithelial intestinal cell viability and the effect on the cell cycles and growth factor signaling were then examined, followed by ER stress-related programmed cell death. The results confirmed the adverse effects of 4NP, and they warned of the hazardous effects of prolonged exposure of the intestine to 4NP. In addition to the intestine, the liver is the main site for detoxification. Paolella et al. examined the cytotoxicity of 4NP to human liver cells [72]. The cell viability of the hepatoma cell line, the HepG2 cells, decreased after 4NP treatment. 4NP also induced the apoptosis-regulating enzyme caspase 3, and the P53 apoptosis regulator, in the HepG2 cells. 4NP gave rise to an unfolded protein response, and a significant increase in the Grp78 protein levels were detected. Finally, 4NP increased the ROS and decreased the enzyme responsible for the antioxidant [117,118].

## 4. Conclusions

The ER is a crucial cellular organelle that participates in protein secretion and lipid storage. The ER reacts sensitively to EDCs; therefore, ER transmembrane proteins could be a good marker for cell damage. The mechanisms of the ER transmembrane UPR alarming genes, such as ATF6, PERK, and Ire1α, are well-established. Therefore, the evidence of cells under ER stress is relatively easy to assess by immuno-blotting against the transmembrane UPR alarming genes and the transmembrane proteins. Table 1 lists 11 well-known EDCs, and an up-to-date list of references are sorted according to the ER stress regulators. For some EDCs, ER stress regulators such as PERK and eIF2α, are detected solely or with their phosphorylated forms. This review discussed the main mechanisms influencing ER under its stressed phase. In the current era, the exposure to EDC is inevitable, and all human and non-human beings are exposed to EDC continuously. We have discussed, in this review, that the prolonged and repeated exposure to EDCs is related to ER stress and the related diseases [17]. We also provided the evidence for EDCs as a trigger for ER stress; however, the basal molecular mechanisms regarding EDCs and the ER are still lack. The clarification of the basal molecular mechanisms could open a new window for therapeutic purposes. Along with this topic, there are few articles that discuss the alleviation of ER stress [31,63], which could also be a new possibility for curing many related diseases. Further research on the field, in thisregard, is required.

## Figures and Tables

**Table 1:** Common EDCs and ER stress inducers.

Chemical	Uses	Classification	Upregulating ER Stress Regulator							
			ATF6	PERK	p-PERK	eIF2α	p-eIF2α	CHOP	Ire1α	GRP78
Atrazine	Herbicide	Hepatotoxicity damage [30], potentially toxic to immune system [9]	[30,31]	[31]		[32]	[32]	[32]		[30,32,33]
Bisphenol A	Plastic, flame retardant	Adverse effect on spermatogenesis [34], hepatic fibrosis [35]	[35,36,37,38,39,40]	[36,37,38,41]	[37,38]	[37]	[37,38,42]	[36,37,38,40,42,43,44]	[36,41]	[29,36,37,38,43,44,45,46]
Butyl-paraben	Cosmetic, food, pharmaceutical product	Reproductive toxicant [6,7]	[47]				[47]		[47]	[47]
Chloropyrifos	Pesticide	Alzheimer’s disease, Parkinson’s disease [48]					[49]	[48]	[49]	[49]
Dibutyl phthalate (DBP)	Plasticizer	Lipid metabolism alteration [50,51]	[52,53]		[54]		[52,53]	[52,53,55]		[52,53,55]
2,4-Dichlorophenol (2,4-DCP)	Herbicide	Endocrine disruptor [8]	[56]				[56,57]	[56,57]		[56,57]
Dichloro-diphenyl Trichloro-ethane (DDT)	Insecticide	Pancreatic β cell damage [58]						[58]		[58]
Di(2-ethylhexyl)phthalate (DEHP)	Plasticizer	Type II diabetes [59]	[60,61]	[60,62]	[62]	[62]	[61,62]	[61,62,63,64]	[60,61]	[60,61,62,63,64]
Glyphosate	Herbicide	Reproducible toxicity [65]		[66]	[66]	[66]	[66]			[66]
Lead	Heavy metal	Possible carcinogen [13] Parkinson‘s disease [67]	[68]					[68]	[68]	[68,69]
4-Nonyl phenol	Surfactant Detergent Plasticizer	Intestine damage [70]						[71,72,73]		[70,71,72,73,74,75]

## Abbreviations

Endocrine-disrupting chemical (EDC), endoplasmic reticulum (ER), unfolded protein response (UPR), corticotropic releasing hormone (CRH), gonadotropic releasing hormone (GnRH), thyrotropic releasing hormone (TRH), growth hormone releasing hormone (GHRH), growth hormone (GH), estradiol (E2), progesterone (P4), thyroxine (T4), tri-iodothyonine (T3), World Health Organization (WHO), Environmental Protection Agency (EPA), endoplasmic reticulum-associated degradation (ERAD), nuclear factor-kB (NF-kB), inositol-requiring 1 protein α (Ire1α), PKR-like endoplasmic reticulum kinase (PERK), activating transcription factor 6 (ATF 6), binding immunoglobulin protein (BiP), eukaryotic translation initiator factor 2α (eIF2α), C/EBP homologous protein (CHOP), X-Box binding protein 1 (XBP1), reactive oxygen species (ROS), 4-methyl-2,4-bis(4hydroxyphenyl)pent-1-ene (MBP), butyl paraben (BP), di(2-ethylhexyl)phthalate, dichloro diphenyl trichloro ethane (DDT), (DEHP), mono(2-ethylhexyl)phthalate (MEHP), dibutylphthalate (DBP), 4-nonyl phenol (4NP).
